# U. S. V. M. A.

**Published:** 1898-03

**Authors:** S. Stewart

**Affiliations:** Secretary; 7½ South James St., Kansas City, Kan.


Secretary.

### U. S. V. M. A.

PRESIDENT SALMON has appointed the following local Committee of Arrangements for the Omaha meeting: Dr. A. T. Peters, chairman, Lincoln; Dr. H. L. Ramaciotti, Omaha; and Dr. John Hall, Falls City, Nebraska. The committee have in part outlined the plans for entertaining all visitors, and will spare no effort to make the occasion one of comfort, pleasure, and profit.

The leading subject for discussion will probably be the all-important one of "Meat-Inspection." It is thought the discussion may be profitably considered under the following general divisions:

Reasons for conducting meat-inspection.

Methods of educating municipalities as to the sanitary importance of meat-inspection.

The necessity of consolidation of municipal abattoirs into one establishment.

Slaughter-house inspection.

Retail market inspection.

Members desiring to present papers at the coming convention should promptly notify Secretary Stewart of their intention to do so.

S. STEWART, V.S.,
Secretary.

7½ South James St., Kansas City, Kan.

